# Scoping Review on Maternal Health among Immigrant and Refugee Women in Canada: Prenatal, Intrapartum, and Postnatal Care

**DOI:** 10.1155/2017/8783294

**Published:** 2017-01-22

**Authors:** N. Khanlou, N. Haque, A. Skinner, A. Mantini, C. Kurtz Landy

**Affiliations:** ^1^Faculty of Health, York University, Toronto, ON, Canada; ^2^Centre for Urban Health Solutions, St. Michael's Hospital, Toronto, ON, Canada

## Abstract

The last fifteen years have seen a dramatic increase in both the childbearing age and diversity of women migrating to Canada. The resulting health impact underscores the need to explore access to health services and the related maternal health outcome. This article reports on the results of a scoping review focused on migrant maternal health within the context of accessible and effective health services during pregnancy and following delivery. One hundred and twenty-six articles published between 2000 and 2016 that met our inclusion criteria and related to this group of migrant women, with pregnancy/motherhood status, who were living in Canada, were identified. This review points at complex health outcomes among immigrant and refugee women that occur within the compelling gaps in our knowledge of maternal health during all phases of maternity. Throughout the prenatal, intrapartum, and postnatal periods of maternity, barriers to accessing healthcare services were found to disadvantage immigrant and refugee women putting them at risk for challenging maternal health outcomes. Interactions between the uptake of health information and factors related to the process of immigrant settlement were identified as major barriers. Availability of appropriate services in a country that provides universal healthcare is discussed.

## 1. Introduction

Canada receives a significant number of newcomers each year that include immigrants, refugees, and asylum seekers. Of the 33 million people living in Canada, 6.7 million (20.6%) are immigrants and 3.5% of the total population are recent immigrants, while over 20,000 women arrive as approved refugees [1]. Additionally, the approximately 8,000 asylum seekers pursuing in-land claims for refugee status in Canada represents an exponential growth in the past decade [1]. The characteristics of all these migrants have changed dramatically as well. In the past fifteen years, there has been a doubling in the proportion of women migrating from source countries such as the Philippines, India, Iran, Nigeria, Iraq, Syria, Columbia, and Eritrea [2]. These same statistics show that a growing proportion of these women are within prime childbearing age and fleeing stressful circumstances (i.e., community violence, war conflict, trauma, or chronic poverty). These changing patterns of migration have implications for both the planning and the delivery of accessible and effective maternal health services, as well as the development of clinical practice guidelines specific to pregnancy in Canada. A scope of literature on maternal health during the pregnancy and postpartum phases is required to establish a foundation of evidence-based findings that can aid the development of guidelines and new policy to better support maternity health.

We report the findings of a scoping review we conducted to examine and outline the extent, range, and nature of empirical evidence about maternal health and healthcare services for migrant women in Canada, from pregnancy through to postpartum. We then provide a thematic analysis according to prenatal, intrapartum, and postnatal care literature for women resettled and living in Canada. The Canadian context allows for a unique examination of the challenges to maternal healthcare due to the highly diverse range of ethnocultural groups and their experiences from premigration and resettlement in a country that provides universal healthcare. For our scoping review, we included all migrants: immigrants who apply to live in Canada typically for occupational or family reunification purposes as well as resettled refugees who are provided with permanent residence and legal status in Canada prior to arriving and migrants who land in Canada with no prior status, asylum seekers, who remain undocumented, without legal status. In Canada, immigrants and resettled refugees are provided with the same universal health insurance that Canadian citizens receive, while refugee claimants are provided with only partial coverage through the Interim Federal Health Program (IFHP) and failed refugee claimants or undocumented migrants receive no health coverage. Unlike other high-income countries with universal health coverage, Canada does not offer medication coverage or exemptions for pregnant women in order to receive health service support regardless of their migration status. Complicating this scenario is the fact that Canada's IFHP health coverage for refugees can be unstable (cancelled at times due to alternating political agendas). Most provinces in Canada also provide funding for midwifery services to immigrants and some refugees. Analysis of studies conducted in Canada reflecting this mix of health services and migrant needs provides a unique opportunity to understand contributing factors to maternity health for migrant women, especially when, for most of them, financial access is not a factor.

Evidence presented in a United Nations report [3] from other similar migrant receiving countries (European Union and Asia) and a recent systematic review of five countries [4] identified a number of factors that lead to underutilization of maternal health services by immigrant women. These include linguistic barriers, the immigrants' level of integration into mainstream society, experience of discrimination and racism from healthcare providers, women's knowledge regarding maternal healthcare information, and recent immigrants' stress of adjusting to a new country. Findings in the UN report also indicate that pregnancy-related problems are common among immigrants throughout the European Union countries and Asia and include maternal health issues such as inadequate or no antenatal care and higher rates of stillbirths and infant mortality as well as financial barriers [5]. Research from the United Kingdom on perinatal outcomes among their immigrant population has reported lower birth weights among babies born to Asian immigrant women and higher perinatal and postnatal mortality rates among Caribbean and Pakistani immigrants as compared to the general population [6]. These findings have also been reported among other high-income countries like Norway, Japan, and Italy [4].

Given the essential nature of these negative perinatal health outcomes in migrants and Canada's unique position of great ethnocultural diversity, new knowledge on the maternal health among migrant women in Canada is warranted to inform care during all phases of maternity. In addition, while guidelines have been developed in Canada for immigrants and refugee healthcare in general, no practice guidelines exist specific to pregnancy, and the current guidelines only include recommendations related to contraception and pregnancy screening [7].

Over the last fifteen years, in particular, as the drive for evidence-based practice has increased, so has the rapid growth in reviews of the literature on specific maternity topics [6, 8–11]. Scoping reviews that use Arksey and O'Malley's [8] methodology help us understand the research landscape by examining the extent, range, and nature of research evidence in a particular research area such as immigrant maternal health [12]. This methodology provides an overall summary of the topic area while also identifying gaps in research knowledge without delving into the types and quality of the studies. This kind of review can form the basis for more detailed reviews [13]. The findings of such scoping reviews can also be used to influence policy and practice.

## 2. Methods

We applied Arksey and O'Malley's [8] framework to explore the large amount of maternal health literature, including both qualitative and quantitative studies as well as previously published systematic reviews to address our research question. Arksey and O'Malley's framework outlines five stages for conducting scoping reviews. These stages are (1) identifying the research question, (2) identifying relevant studies, (3) study selection, (4) charting the data, and (5) collating, summarizing, and reporting the results and (6) an optional step of a consultation exercise is suggested by Arksey and O'Malley to validate the findings from the scoping review. The five stages of the framework may appear to be linear; however, the actual process and stages are iterative and reflexive [8]. The flexibility of this methodology allowed us to redefine our search terms as our familiarity with the literature increased [8], thus ensuring comprehensive and broad coverage of the literature. The format and the pursuing subheadings of the paper follow the recommendations of Arksey and O'Malley [8] and others [9, 11] to guide our reporting and maintain transparency of our work.

The research question of our scoping review was the following:* What does the peer-reviewed scientific literature tell us about maternal health and health service utilization during the prenatal, intrapartum, and postnatal periods among migrant women resettled in Canada?* We were guided by the World Health Organization's (WHO) definition of maternal health. WHO defines maternal health as “the health of women during pregnancy, childbirth, and the postpartum period” [5]. We also utilized the definition of “immigrant” proposed by the Canadian Council of Refugees: “a person who has settled permanently in another country (Canada)” [14], but we restricted our target population to female immigrants identified as being first generation and foreign-born. Further, to properly reflect the current trend of diversity in migration, we included refugees with legal status as well as asylum seekers seeking formal refugee status and failed refugee claimants or women with undocumented status or no legal status.

### 2.1. Search Strategy

A scoping review of studies reporting health outcomes and utilization of healthcare services for migrant women living in Canada and related to pregnancy and postpartum outcomes was conducted. A literature search for relevant articles published between 1 January 2000 and 1 September 2016, without methodological restrictions, was carried out in the electronic databases PubMed, CINAHL, PsycINFO, Ovid MEDLINE, Science Citation Index, the Social Sciences Conference Proceedings Index, and Sociological Abstracts (2000–2016). However, articles were restricted to those published in English and in peer-reviewed scientific journals for studies on pregnancy and childbirth health outcomes and healthcare utilization for migrant women, using the search terms outlined in Table 1. These electronic databases were chosen as they contained studies relevant to our research question and the search strategy was designed according to the specification of each database. Originally, we had fewer search terms but, later, based on our knowledge gained from the literature, we included additional terms such as birth spacing, family planning, breastfeeding, and violence to be more comprehensive. The search terms (Table 1) were performed alone and in combinations using the Boolean operators “AND” and “OR.” There were no differences in the databases in terms of results yielded, but broad terms such as “immigration” or “refugee health” were excluded as search terms on their own, to allow for inclusion of articles that were specific to pregnancy and childbirth among migrant women living in Canada. The last search was carried out on 1 September 2016 and searches were conducted in consultation with a health librarian and the research team and entered [15, 16].

To answer the research question of our scoping review, we used the following inclusion and exclusion criteria.

#### 2.1.1. Inclusion Criteria

Inclusion criteria were (i) primary and secondary source research studies, including systematic reviews, published in English language, between the years 2000 and 2016; (ii) all studies (qualitative, quantitative, and mixed methods) which included migrant women as the sample population and focused on maternal health (i.e., prenatal, delivery, perinatal, or postnatal period); (iii) studies that had migrant women living in Canada as participants.

#### 2.1.2. Exclusion Criteria

Exclusion criteria were (i) the grey literature, documents published in non-English languages, theses, protocols, policy documents, proposals, and editorials and (ii) non-peer-reviewed publications.

One research team member initially read the titles identified in searches on the electronic databases to determine the relevance of the study to our scoping review. Titles that did not contain the search words in the title field were reviewed and removed if they did not meet the inclusion criteria. Three members of the research team then reviewed all the abstracts to select the full-text documents to be included in the scoping review.

## 3. Results

**Figure 1:**
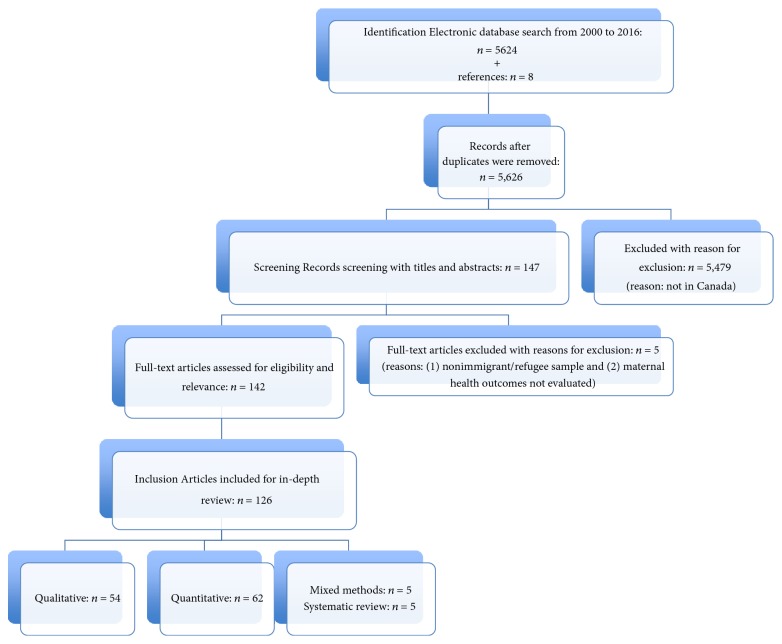
Flowchart illustrating the scoping review process.

A total of 5,608 studies were identified in our initial search based on the search words and an additional 8 studies were added after identification from references of these articles. After the removal of duplicates and removal of all studies that did not meet the inclusion criteria within the body of the article, we were left with 147 studies. Of these, another thirteen articles were removed because they were not specific to the Canadian population or because they were study protocols with no results. As a result of this process, 126 documents were included for full-text review and charting (Figure 1).

Of the 126 studies included in the final synthesis, 54 were qualitative, 5 were mixed methods studies, 5 were systematic reviews, and 62 were quantitative studies, and only 3 of the research studies were intervention studies. Among the 54 qualitative studies reviewed, several stand out as focused on the diversity of the women: 6 focused on childbearing immigrant women from a specific source country, such as Haiti, Vietnam, or Pakistan or countries within the Middle East [6, 17–21], while three studied new immigrant mothers settled in Canada for less than 5 years [22–24], one sampled recent refugees [25], and three examined women identified as uninsured asylum seekers [17, 26, 27]. Similarly, within the 62 quantitative studies, 18 studies in particular focused on specific ethnic groups, including 3 longitudinal studies of health outcomes [26, 28, 29], another three studies focused on mental health concerns during the prenatal period [30–32], while 2 evaluated mental health status during delivery [33, 34], and another 3 studied physical status at various periods of maternity [35–37]. Of the five mixed methods studies, 2 focused on the role of culture in the prenatal period [10, 38], another two evaluated mental health also during the prenatal period [39, 40], and one final study examined understanding of procedures during delivery [41].

Results are presented according to the three main subgroups: (1) prenatal care (migration status, postmigration factors, and health services factors), (2) intrapartum care (maternal and child health), and (3) postnatal care (healthcare access and individual factors). The number of documents retrieved from each of the electronic databases as well as the number of studies related to outcomes, population, and methodology is listed in Table 2.

### 3.1. Factors Associated with Prenatal Health Outcomes

In total, 20 studies focused on maternal health outcomes during the prenatal period for immigrant and refugee women. Twelve studies examined migration and maternal lifestyle factors in relation to maternal health and five studies examined women's mental health concerns as a contributing factor during the prenatal period.

#### 3.1.1. Migration Status

Studies that examined Canadian immigrant women found no differences in their ability overall to navigate or initially access the health system for prenatal care which was generally measured by the number of medical appointments during pregnancy [18, 42]. However, when the adequacy or quality of prenatal care was specifically studied, over 20% of immigrant mothers who were new to Canada were found to have received “inadequate” prenatal care leading to identification of mother's ethnicity as a risk factor [18, 42]. Prenatal care in uninsured asylum seeking refugees or undocumented women was found to be inadequate [17, 38, 43, 44]. Wilson-Mitchel and Rummens reported that as many as 80% of these uninsured women receive less than adequate prenatal care and 6.5% receive no prenatal care at all [38].

Language barriers and limited cultural sensitivity of prenatal care may also interfere with established guidelines. Language was most commonly cited as a barrier to care [21, 45–49]. For many immigrants, English as a second language makes it difficult for them to understand the new healthcare system and many of the medical terminologies commonly used by providers. They also face difficulty in effectively communicating with service providers. Cultural factors were perceived to be a barrier to accessing prenatal care both by immigrant women and by the healthcare providers who want to support them. Cultural needs and expectations are often unknown or unable to be met in the context of the Canadian healthcare system [21, 44, 46, 48–50], which may negatively impact the experience of immigrant women and lead them to cease or turn elsewhere for care [46, 50]. Bhagat and her colleagues [50] found that female South Asian immigrants were often not comfortable expressing their needs or identifying gaps in the system [51].

#### 3.1.2. Lifestyle Factors

Immigrant women were less likely to smoke and consume alcohol during pregnancy [18, 52] with a positive correlation existing between length of stay in Canada and alcohol and tobacco consumption before pregnancy [53]. Kowal et al. [53] found that immigrant women gained less weight during pregnancy than Canadian-born women and were 1.5 times more likely to gain less than the Health Canada recommended amount [54]. Another study by Hyman and Dussault [19, 40] found that acculturation was correlated with increase preoccupation with thinness, even during pregnancy, but did not examine weight gain. A number of in-depth studies by Higginbottom and her colleagues [19] have consistently found food choices to correlate with cultural beliefs, and, despite recommended health guidelines, immigrant women may continue to eat culturally preferred foods despite medical advice. This finding was also highlighted in a previous literature review of studies between 2000 and 2010 [55] which also identified the need for increased cultural knowledge relating to food choices and the need to involve the whole family in decisions related to food and nutrition.

#### 3.1.3. Mental Health Status

Migrant women are more likely to suffer from prenatal depression than their nonmigrant Canadian counterparts [20, 33]. Women who were immigrants experiencing prenatal depression were more likely to report suffering from somatic symptoms [33]. Some of the identified risk factors associated with prenatal depression in migrant women included high marital strain [20], lack of social support, poverty [20, 30], and crowding [20] and migrants were also found to demonstrate elevated maternal cortisol levels [31]. Migrant women from certain countries may be more at risk than those from other countries. Miszkurka et al. [20] found that immigrants from the Caribbean, sub-Saharan Africa, and Maghreb experienced a twofold increase in antenatal depressive symptoms when compared to women who immigrated from Europe and the Middle East. It is important to note that their risk attenuated when social support and financial situations were accounted for [30]. Also very noteworthy is the finding that immigrant women were less likely to report experiencing violence associated with pregnancy [28, 55–57]. Stewart et al. [29] found that the risk of violence associated with pregnancy increased if the migrant was an asylum seeker, lived in Canada for less than 2 years, lived without a partner, or had less than high school education. In these women, violence during pregnancy was also associated with a higher risk of prenatal depressive symptoms, with victims of intimate partner violence experiencing a 3- to 5-fold increase in depressive symptoms.

#### 3.1.4. Physical Health Status

 McElroy et al. [58] examined rates of rubella immunity in immigrant populations, as underimmunized populations were at increased risk for congenital rubella syndrome. Rubella immunity was found to be the lowest among women from North Africa and the Middle East and China and the South Pacific [58]. Immigrant women were also less likely to take folic acid supplements before and during pregnancy, reporting that they did not have enough information about the benefits [32]. When the effect of the Canada Prenatal Nutrition Program (CPNP) on the health behaviours of immigrants and Canadian-born women was examined, the CPNP was found to be effective in increasing vitamin use and breastfeeding in immigrant women, while decreasing their consumption of tobacco and alcohol products [35]. Immigrant women showed the strongest correlation between CPNP exposure and positive health behaviours while refugee women tended to not take supplements, likely because they also did not have visits with their community public health nurse or sufficient prenatal appointments with a physician.

### 3.2. Factors Associated with Utilization of Health Services during the Prenatal Period

Factors hindering access to care were identified in terms of navigating services, mothers' perception of quality of care, socioeconomic status, language, and cultural and other barriers. As many new migrants are not familiar with the organization of the healthcare system, accessing healthcare and understanding the role of different services and healthcare providers act as a barrier to seeking care and accessing services [21, 43, 46, 59].

Many immigrant women feel as though they are receiving substandard care if their prenatal care is not overseen by a medical doctor [21, 46] and have obstetricians overseeing their prenatal care at rates higher than Canadian-born women [21, 32]. Some immigrant women also try to utilize multiple or different care providers during pregnancy [46] or seek alternative supports, including private services [18, 21] which may affect the quality and continuity of care they receive. Heaman and her colleagues [60] examined the relationship between prenatal care and neighbourhood context and found greater levels of inadequate prenatal care in neighbourhoods with higher proportions of recent immigrants. The at-risk neighbourhoods also had the highest rates of unemployment, women who reported smoking during pregnancy, and the highest percentage of single-parent families and individuals with less than 9 years of education [60]. Past reviews have also found the less favorable socioeconomic status of immigrant women to compromise their access to prenatal care [21]. Findings also demonstrate other important barriers affecting care, including finding a doctor who could speak their own language [21, 43, 45], lack of transportation [48], weak social support [49], need for childcare [48, 50], financial barriers [43, 44, 50], and having lost their health records during migration [45]. Fears of reporting to immigration authorities and deportation were also considered to be barriers for undocumented pregnant women [21]. Uninsured women that also presented for care later in pregnancy had fewer prenatal visits and underwent less auxiliary testing than insured immigrant women [17, 38, 43, 61]. Acculturation into a new country of residence is a process that takes place over time and is influenced by personal beliefs and behaviour as well as general integration receptivity of the host society. As a result, ethnocultural or traditional practices may interfere with the recommendations provided to migrant women by Canadian health service providers for optimal health of mother and child. Low levels of acculturation were consistently found to be a barrier to prenatal care among immigrant women living within inner-city neighbourhood [62].

Migrant women were less likely to attend prenatal classes than Canadian-born women [32, 47]. They were also found to express a poor understanding of the purpose of prenatal monitoring, namely, symphysis-fundal height measurement, gestational diabetes screening, and Group B* Streptococcus* testing [47, 63]. A study by Bhagat and colleagues [50] examining the maternal experiences of Punjabi women suggested that the term* classes* poses a problem, as this was said to imply ignorance towards childbirth. Other barriers associated with the lower rates of immigrants attending prenatal classes were language [47] and time constraints as their need to earn a living wage often took precedence over healthcare [50].

### 3.3. Factors Associated with Intrapartum Health Outcomes

In total, 35 studies were found on intrapartum care. Of these, 20 studies focused exclusively on delivery care and 15 studies on prenatal as well as intrapartum care. Several studies also pointed out specific concerns for migrant women related to eclampsia, caesarean section, delivery experiences, birth weight, and perinatal mortality, as well as preterm birth. During the intrapartum period (occurring during labor and delivery), migrant women expressed difficulties with communication and integration of their cultural beliefs with recommended healthcare practices, as well as lack of support from healthcare providers and lack of understanding of the informed consent process for procedures during delivery [25].

#### 3.3.1. Delivery Experiences

Several studies examined the delivery experiences of immigrant women. Overall, migrant women do not exhibit higher rates of severe morbidity; however, disparities in severe maternal morbidity have been identified for refugee women. This is especially true now that immigration trends favour humanitarian protection for refugee women with HIV [64]. Similarly, immigrant women from sub-Saharan Africa are consistently found to be at higher risk of severe maternal morbidity with the most common diagnosis being eclampsia followed by uterine rupture [49]. With regard to eclampsia, in particular, however Ray et al. [25] found a positive correlation between eclampsia in immigrant women and the number of months since immigration. Urquia and his colleagues [34] found that immigrant women from the Caribbean and Hispanic America are also at a higher risk of severe preeclampsia than locally born populations or immigrants from industrialized nations.

Conflicting data exists regarding the incidence of caesarean section in immigrant populations. Some studies have found that immigrant women were at a significantly higher risk of caesarean section [18, 38, 65, 66], while others have reported no difference in caesarean section rates between immigrants and Canadian-born women [17, 32].

Maternal source country of origin was identified as a predictor of caesarean section in two studies [36, 67]. In particular, these studies found that Vietnamese and Eastern European women underwent the fewest caesarean sections while immigrant women from Africa, South America, and South Asia exhibited the highest rates of emergency caesarean sections [36, 67]. Differences by migration status also varied for immigrant women, with refugees from South East and Central Asia having the highest rates of caesarean section.

Conflicting data exists for the incidence of preterm birth among immigrant women. Some studies found no difference in preterm birth for immigrant mothers or uninsured refugee or undocumented women [17, 38, 65]. However, when specific source countries were studied, immigrant women from Guyana, Trinidad and Tobago, Philippines, and Jamaica were all found to be at higher risk of “very preterm babies” [68]. Some studies found that migrant women from Asia and sub-Saharan Africa as well as Haiti were at a greater risk of preterm birth [69, 70]. Undocumented migrants and asylum seekers were also found to have an increased likelihood of preterm birth [18, 44]. In most of these studies, length of time in Canada was associated with equal or decreased incidence of preterm birth [42, 69, 71–73]: risk of preterm birth increasing with a longer stay in Canada [42, 73].

Residential environment, including neighbourhood poverty, was found to have little or no association with preterm birth in immigrants [22, 23, 72] but, interestingly, a high same-ethnic group concentration was associated with low birth weight [22]. On the other hand, a high density of migrant population was found to have a protective effect against preterm birth in immigrant mothers, especially those with higher education [23].

The link between education and adverse birth outcomes remains unclear. Auger found no correlation between low education and adverse birth outcomes (low birth weight, preterm birth) in migrant women but found that foreign-born status was associated with preterm birth in university educated women [74]. Higher education was similarly found to be more protective against preterm birth for Canadian mothers, while immigrant mothers required up to four years more education to experience the same protective effects [75]. Battaglini and her colleagues reported that women at medium risk for perinatal problems also had higher education but they were also more likely to have undergone professional dequalification as a result of their immigration to Canada [76].

#### 3.3.2. Birth Weight and Perinatal Mortality

Recent immigrants have been found to be at a higher risk of having low birth weight (below the 10th percentile) infants [65]. Immigrant women in Canada were more likely to have low birth weight infants and those infants were more likely to be classified as having a low birth weight [22, 77–80] based on Canadian birth weight curves. This is especially true for immigrant women of East or South Asian descent; however, a number of researchers have discovered that when region-specific birth weight curves from the mothers' country of origin were used, this higher risk disappeared [22, 78, 81–83]. Some of these studies [22, 81, 83] have also found that infants of migrants were more likely to be misclassified based on Canadian birth weight curves, suggesting the need for ethnic specific, culturally sensitive standards [77, 84]. Confirming this conclusion is that Chinese and South Asian infants had lower perinatal mortality risks throughout gestation, despite having lower birth weights [78]. At the same time, a positive correlation between violence associated with pregnancy and low birth weight infants in immigrant women has been noted [29]. In contrast, a more recent systematic review by Boshari and his colleagues found that the newborns of migrant mothers had higher birth weights than the infants in their native countries [37]. In contrast, Gagnon and her colleagues [36] found that the infants of Asian, North African, and sub-Saharan African migrants were at higher risk of feto-infant mortality. Migrant women who experienced violence associated with pregnancy were at higher risk of miscarriage [29, 85]. Infants of uninsured asylum seeker mothers required more neonatal resuscitation [18, 38].

### 3.4. Factors Associated with Utilization of Health Services during the Intrapartum Period

Almost no studies have been published that examine utilization of health services during the intrapartum period, with a few exceptions. Chalmers and Hashi looked at the birthing experiences of Somali women in Canada, with a history of genital mutilation, and found many reports of inadequate pain management and cultural insensitivity [86]. The women reported feeling left out of decision-making regarding their care and undergoing more medical interventions than they had hoped for. The women reported experiencing hurtful comments, apparent disgust, and a lack of privacy during procedures. Women expressed the importance of cultural sensitivity and awareness during this process [49, 86–88], a need which some women felt was not met in the context of the western healthcare system [49, 86, 89]. Many women expressed a preference for a female physician during the labor and delivery process [39, 49]. When compared to Canadian women, migrant women were more likely to rate their birth experience as “somewhat positive” instead of “very positive” [32]. Several migrants did not support medical interventions during pregnancy and instead expressed a desire to withstand the labor and delivery process without the use of pain medications [37, 39, 90].

### 3.5. Factors Associated with Postnatal Health Outcomes

In total, 71 studies focused on maternal health outcomes during the postnatal period for immigrant and refugee women. Most of these studies have been published more recently and are highly consistent in their findings, providing evidence for increased risk of maternal mental health challenges and negative health trajectories from cultural barriers following the birth of a child.

#### 3.5.1. Mental Health Outcomes

All studies of the postnatal period for immigrant women discussed postpartum depression and several studies identified both cultural background and socioeconomic factors as contributors to postpartum depression in migrant women [41, 85, 91–95]. The stigma of emotional distress, experiences of increased discord within family relationships, and the difficult external expectations of motherhood from the society, as well as poor nutrition and self-care practices during this period of maternity, are consistently cited as leaving migrant women vulnerable to postpartum depression [41, 93]. Further economic dependence and insecure migration experiences also disadvantage migrant women [93, 96]. Two recent longitudinal studies of immigrant women found that younger women who had been in Canada for less than two years and those who had poor perceptions of their own health and low income exhibited a high prevalence of postpartum depression [93, 95]. In addition, poor use of supplements, inadequate nutrition, and reduced self-care during the postnatal period have been documented to also contribute further to poor maternal health outcomes.

#### 3.5.2. Resilient Coping

One study that investigated the impact of postnatal stress on immigrant women's adjustment, months and years following delivery, found that immigrant women typically responded with resilient coping strategies [96]. Gagnon and her colleagues interviewed a group of immigrant women in Toronto and Montreal, deemed to be at high risk in their postnatal period due to factors discussed above but to be adjusting well without any maternal health concerns. Women categorized as “resilient” identified social inclusion through a socially supportive network, accessible health services, and culturally sensitive interventions as experiences enhancing their coping strategies [96].

### 3.6. Factors Associated with Utilization of Health Services during the Intrapartum Period

Nowhere is the negative impact of poor access to healthcare reflected more than in the immigrant woman's higher incidence of postpartum depression, reported to be at least twice the rate of nonimmigrant women [97]. It is not surprising that barriers to care such as lack of acculturation or language barriers and culturally appropriate care begins in the prenatal period and persists through to the postnatal period, but it is surprising that other factors including family pressures and personal adjustment needs can create situations in which barriers and risk to maternal health are intensified for immigrant women in the postnatal period. Whether the needed care is for mental health, their child's well-being, or their own physical health, studies indicate that some postnatal outcomes associated with maternal health for immigrant women are highly related to their experiences of isolation, poor family relationships, conflicted gender roles, and the discrimination they perceive within health system services [26, 41, 92, 97]. The immigrant participants of a community-based participatory research study represent their challenge best: that, as immigrant women in Canada, they “lacked the social and environmental factors perceived as key enablers of healthy pregnancies and postpartum” [27]. As the immigrant women's postnatal outcomes include higher rates of postpartum depression (PPD) [93, 95] and lower rates of breastfeeding exclusivity in immigrant women with higher levels of depression symptoms [94] and stress related physical conditions [97], their experience with mental health through the postnatal period is an important area of consideration for immigrant and refugee women.

#### 3.6.1. Isolation and Discrimination

The existing communication problems, inadequate information, and lack of familiarity with healthcare systems persist into the postnatal period, but more intensified are immigrant mothers' perceptions of discrimination and care which they perceive as not kind or respectful [61]. Consistently, findings demonstrate the negative impact of family and community experiences on immigrant women's postnatal health. They are required to cope concurrently with migration stressors and new parenthood, both contributing to higher levels of loneliness and stress that is further exacerbated by time restrictions for financial support, prolonged family reunification processes, and uncoordinated government services that serve the postnatal period [26]. Further supporting increased stress experienced by asylum seekers or undocumented migrant women, family physicians reported caring for this population to be challenging, increasing their workload and psychological stress [61].

#### 3.6.2. Poor Family Relationships and Gender Roles

When women's perceptions and experiences during the postnatal period are explored, a clear association between women's maternal health and family relationships is identified [26, 92, 95]. Not only do they have to cope with the social stigma of being in need, being under stress, and being lonely [26], but also their decisions about healthcare are influenced by relationships with parents and their in-laws [41]. Marital discord during the postnatal period is also reported to affect a migrant woman's access to healthcare services [93]. Adding further to their experience of postnatal stress is the experience of many complex gender-related problems [41, 93]. Gender based expectations within their family, marital discord evoked by new gender roles, and the conflict with their newly developing identity within the Canadian society intersect with cultural and social norms in a way that hinders women's ability to access necessary healthcare services [26, 93].

## 4. Discussion

This scoping review has confirmed the existence of a series of cultural, social, economic, and individual factors that relate to migrant health and which create barriers to maternal health throughout the prenatal, intrapartum, and postnatal periods of maternity for women living in Canada. Several of these factors appear to serve as potential barriers to healthy pregnancy and birth outcomes, while others appear to interact in a complex manner to affect maternal health outcomes. The information derived from this review helps healthcare providers or social care professionals in Canada understand the complexity of migrant women's maternal health experiences, leading to improved interventions, practice, and policy.

The literature indicates that migration and resettlement experiences influence maternal health throughout pregnancy, delivery, and the postpartum period. While optimal health during all three periods of maternity is a priority, meeting this goal is a challenge for migrant women. Even in Canada, where universal healthcare exists, financial barriers have been identified, including those that intersect with migration status, stress of acculturation, and other postmigration experiences. In addition, migrant women's dependence on family and social support puts them at risk. However, while some ethnocultural factors demonstrate a negative impact on maternal health outcomes for migrant women, others foster enhanced resiliency. Given the tremendous heterogeneity of migrant women in Canada, the consistency of barriers and maternal health outcomes perceived and experienced is noteworthy.

Similar barriers to utilizing health services impacted the three periods of maternity, at varying degrees, but health outcomes differed. During the prenatal period access barriers to maternal healthcare services may disadvantage some migrant women. However, the findings in relation to intrapartum outcomes are not as consistent across studies. For instance, factors contributing to health outcomes during the prenatal care are influenced by those typically categorized as social determinants of health [98]. On the other hand, contributors to postnatal health outcomes are influenced by more specific factors occurring within the women's personal lives. Migration is increasingly recognized as an important social determinant of health and intersects with factors such as downward socioeconomic mobility, poor access to optimal nutrition, and limited social networks [61, 96, 99]. In addition, barriers to services further disadvantage pregnant migrant women and appear to also contribute to at-risk maternal health outcomes, including postpartum depression [93, 95] and stress related physical conditions [97]. On the other hand, factors related to intrapartum care influence the adequacy and effectiveness of maternal health services. Canada has a publically funded healthcare system that is believed to provide equal access to the quantity of medically required hospital and physicians' services [54] but inconsistent access to midwifery and no clinical obstetrical guidelines for migrant women [100] affected both health outcomes and the experience of barriers to care for migrant women. Inconsistencies emerging in the literature around intrapartum care may be related to variations across settings in the effectiveness of intrapartum services for immigrant women.

Another reason for the inconsistency in findings across studies is that immigrant women are a heterogeneous group but the current review demonstrates an interaction with barriers and health outcomes depending on the period of maternity. The unique ethnic, cultural, and socioeconomic factors, language, family makeup and size, and quality or need of social networks can exert important and synergistic influences on maternal health and healthcare experiences [5, 32, 38]. The current review supports the call for improvements in maternal health outcomes and healthcare utilization through consideration of those factors that are unique to migrants, including the woman's country of origin and the particulars of migration experience for the women. In addition, the category of migrant may be used inconsistently across studies and entail different migration statuses such as refugees or landed immigrants, each with their own unique resources and barriers to services, yet the review demonstrates high levels of consistency in the existence of barriers to utilizing maternity care. Additionally, government-assisted refugees in Canada are recognized as Convention Refugees abroad and have assistance from the government during their initial resettlement period that includes both healthcare and family or community supports [101] while migrants who are applying for legal status in Canada have healthcare insurance but no family/community support [102]. Further, those migrants who have given up trying to obtain legal status do not have healthcare coverage or any other supports. Migrants who remain undocumented are likely to have experienced trauma and ill health [44] and have to cope with greater variability in the precarious nature of their lives based on the circumstances of their arrival into Canada as well as the stress of deciding what to do with newborns of migrants who become parents while waiting in Canada for their asylum claim to be settled [25, 66, 78]. In all, the literature is fairly consistent in identifying significant risks to asylum seeking women and also for refugee and immigrant pregnant women.

Our findings corroborate the more recent systematic reviews of studies on immigrant and nonimmigrant maternity care [14, 37, 55, 67, 97, 99, 103]. In particular, Small and her colleagues [103] evaluated studies on maternal healthcare across 5 countries (Australia, Canada, Sweden, the United Kingdom, and the United States). This team concluded that while migrant and nonmigrant women have similar hopes from maternity care, migrant women provide poor ratings of care received and face additional challenges that impact negatively their health throughout all three phases of maternity. Of significance, Small and her colleagues conclude that while academics often recommend culturally sensitive care, immigrant women want care that is respectful and communicated well and offers specific information on maternity procedures in their community [103]. In addition, they call for enhancing equity and nondiscriminatory attitudes in care [97, 103]. In our scoping review, the challenges reported across studies associated with negative impacts on immigrant women's experiences in accessing prenatal care included navigating the healthcare system, understanding how care and provision of consent operate in their new homeland, addressing language and communication challenges, finding a doctor who could speak their own language, lack of attention to cultural concerns, cost of services, and perception of poor quality of care, often due to a discrepancy between care provider recommendations and the traditional expectations of the mother, husband, and their parents. Studies also noted that migrant women were less likely to smoke and consume alcohol but also less likely to attend prenatal classes and report violence during pregnancy, and they also demonstrated lower rates of immunization, nutritional supports, and supplements uptake during pregnancy. Migrant women were also found to be much more likely to suffer from both prenatal and postnatal depression which was also found to negatively impact their levels of nutrition and self-care. In addition, our scoping review supports two important current trends in migration, including a dramatic increase in female migrants, and the case for very specific factors that contribute to inequity in migrant women's maternity care, which can best guide policy.

## 5. Limitations of Scoping Review

Our scoping review has two limitations that need to be taken into account. First, this review did not look into grey and unpublished literature. Second, the results of the review should be interpreted with caution because scoping reviews do not screen for quality of studies and, thus, have large variations in study methodologies and sampling of the studies included in the review.

## 6. Conclusion

Pregnancy and childbirth for migrant women living in Canada carry a high risk of adverse health and mental health outcomes. Utilization of healthcare can also be compromised for migrant women who are pregnant, despite the availability of Canada's universal healthcare system. Both health and utilization outcomes further put migrants with no legal status, as in the case of asylum seekers, at risk. Consistent with the findings of migrant women in European and western countries as a whole [104, 105], our scoping review has identified a consistent body of knowledge supporting these challenges in Canada as well, where financial access is not typically a barrier and where migration policies are generally believed to be inclusive. However, gaps in knowledge are evident and more importantly, at this time, there are no clinical or scientific guidelines in obstetrics that consider the health of migrant pregnant women. By integrating studies and thematically analyzing their findings, this scoping review has created a solid foundation for recommendations that can lead to research, practice, and policy improvements in all countries facing increased diversity of women migrants and that already have broad healthcare coverage.

In light of the scoping review findings, the following recommendations are made for future research, practice, and policy:

1. Disentangle effects of ethnic and immigration contributions to maternal health through comparative research designs including migrant and Canadian-born women with diverse identity and cultural and lifestyle markers.
2. Focus on targeted intervention studies that can provide specific evidence for scaling up maternal health programs based on translation of knowledge regarding nutrition, procedures, and health practices, especially for the prenatal and postnatal periods where women migrants are at the greatest risk.
3. Include mothers in the study prior to actual data collection to inform study design (such as through participatory approaches to research) or after the findings for dissemination to determine relevance of findings for diverse communities. This is important for clinical practice as well to ensure that providers and decision-makers understand how to individualize services to best meet the needs of pregnant migrant women.
4. Engage in knowledge transfer and mobilization informing studies to help understand how interactions between service providers and mothers with few socioeconomic resources or individual family and community factors affect migrant mothers' utilization of services and access to maternity healthcare.
5. Given the higher risk of negative maternal health outcomes for migrant women without legal status, the policy implications of this scoping review are noteworthy. In particular, three provinces in Canada have 3-month wait for health insurance for landed immigrants and sponsored-class immigrants, comprising over 80% of newcomers to Canada [106]. Also, since there is a large increase in migrant women asylum seekers at a time of higher rejection rate for those applying for refugee status, it is likely that more pregnant women are put at risk for poor maternity health outcomes as they also fail to be able to utilize healthcare services.
6. The recommendations that emerge from this scoping review support formulation of policies that reduce barriers and the contributors to poor maternity health outcomes, including language needs, health knowledge, and cultural influences. The thirteen other countries, such as Denmark, Italy, Japan, Norway, and Switzerland, identified as “high-income” countries offer exemptions for pregnant women [107]; Canada does not.
7. The health status and utilization of services by pregnant migrant women depend to a large extent on health education, access to services, and cultural factors, as evident in our scoping review, supporting the need to increase opportunities for improving the cultural communication by providers, especially for subpopulations of migrant women and their pregnancy needs in relation to their personal, culture, and family needs.
8. Internationally, healthcare systems for reproductive/maternal services are prioritized to decrease maternal morbidity, but our scoping review indicates limitations in Canada and also supports the identified complexities in the lives of migrating women, including spousal relations, community roles, and self-care needs, demonstrated as critical to their health status and utilization of healthcare services to optimize pregnancy and delivery. Policy that directs a more integrative model of maternity care is needed to help maternity healthcare providers and researchers include the multidimensional experiences and challenges of pregnant women who migrate and the influences on their health.
9. Finally, the current lack of existing clinical practice guidelines cannot continue. Official provisions need to be made by healthcare providers and clinical practice guidelines need to be produced for the care of migrant women during the maternity period, similar to that produced for indigenous populations [100].

## Figures and Tables

**Table 1:** Search terms used.

immigrant prenatal care	refugee maternity
immigrant maternal care	refugee maternal health
immigrant antenatal	refugee antenatal
immigrant postnatal care	refugee prenatal care
immigrant intrapartum care	refugee intrapartum care
immigrant maternal health	refugee postpartum care
immigrant postpartum care	refugee childbirth
immigrant childbirth	refugee postnatal care
immigrant maternity	refugee family planning
immigrant child spacing	refugee intimate partner violence
immigrant family planning	refugee pregnancy violence
immigrant breastfeeding	refugee child spacing
immigrant intimate partner violence	refugee breastfeeding
immigrant pregnancy violence	Maternal health care refugee

**Table 2:** Content of studies and database search.

	Frequency	Percent
*Period of maternity *(*n* = 126)		
Prenatal	20	15.9
Intrapartum	35	27.8
Postnatal	71	56.3
*Migration status *(*n* = 126)		
Immigrant	95	75.4
Refugee	24	19.0
Asylum seeker	7	5.6
*Prenatal outcomes *(*n* = 20)		
Migration	6	30.0
Lifestyle	3	15.0
Mental health	6	30.0
Access & utilization	5	25.0
*Intrapartum outcomes *(*n* = 35)		
Complications	3	8.6
Ethnicity	4	11.4
Birth weight/mortality	8	22.9
Nutrition	8	22.9
Mental health	4	11.4
Access & utilization	8	22.9
*Postnatal outcomes *(*n* = 71)		
Mental health	12	16.9
Resilient coping	18	25.4
Discrimination	11	15.5
Poor relationships	17	23.9
Access & utilization	13	18.3
*Social determinants of health*		
Income	9	60.0
Neighborhood	4	26.7
Education	3	20.0
*Methodology *(*n* = 126)		
Qualitative	54	42.9
Quantitative	62	49.2
Mixed methods	5	4.0
Systematic review	5	4.0
*Electronic search databases *(*n* = 5624)		
PubMed	2416	43.0
CINAHL	1320	23.5
PsycINFO	1490	26.5
Sociological & Science	479	8.5
